# Heart Rate Performance Curve Is Dependent on Age, Sex, and Performance

**DOI:** 10.3389/fpubh.2020.00098

**Published:** 2020-04-02

**Authors:** Philipp Birnbaumer, Heimo Traninger, Andrea Borenich, Markus Falgenhauer, Robert Modre-Osprian, Hanns Harpf, Peter Hofmann

**Affiliations:** ^1^Exercise Physiology, Training & Training Therapy Research Group, Institute of Sports Science, University of Graz, Graz, Austria; ^2^ZARG Centre for Outpatient Rehabilitation, Graz, Austria; ^3^Department of Production and Operations Management, University of Graz, Graz, Austria; ^4^Center for Health & Bioresources, AIT Austrian Institute of Technology GmbH, Graz, Austria

**Keywords:** heart rate deflection, ß1-receptor sensitivity, intensity prescription, sex differences, maximal heart rate, aging

## Abstract

**Introduction:** The Heart Rate Performance Curve (HRPC) is neither linear nor uniform and related to ß1-adrenoceptor sensitivity. As aging and exercise influence ß1-adrenoceptors we suggested age, sex and performance effects on the HRPC. Aim of the study was to examine the effects of aging on the deflection of the HRPC in maximal incremental cycle ergometer exercise (CE) in a large cohort of healthy subjects.

**Methods:** Heart rate (HR) data of 2,980 men (51 ± 15 years) and 1,944 women (52 ± 14 years) were classified into age groups (≤20 up to >80 years). We analyzed age and performance (P_low_ 25%-quartile and P_high_ 75%-quartile of age predicted power) effects on HR_max_ and on the degree (*k*) and the type (regular downward deflection *k* > 0.1, linear −0.1 ≤ *k* ≤ 0.1 and atypical upward deflection *k* < −0.1) of the HRPC.

**Results:** *k*-values decreased significantly with age in men and women and were significantly higher in women. Atypical HRPC's increased by a linear trend from ≤20 to 70 years (*m*) respectively 80 years (*w*) from 10 to 43% (*m*) and 9 to 30% (*w*). HR_max_ of all age groups was lower in P_low_ and overall number of atypical HRPC's was 21% (*m*) and 16% (*w*) higher compared to P_high_.

**Conclusion:** Aging increased the number of atypical HRPC's with upward deflection in CE tests, which influences exercise intensity prescription especially when using fixed percentages of HR_max_. Changes in HRPC's were affected by sex and performance, where women generally and subjects with higher performance presented less atypical HRPC's even at older age.

## Introduction

Research in the past few years revealed, that the heart rate performance curve (HRPC) in incremental exercise is neither linear nor uniform (1), which was shown to have an impact on exercise prescription (2). With stepwise increasing intensity, heart rate increases progressively in an s-shaped manner which has already been shown by Brooke and Hamley (3). Later on, Conconi et al. (4) used the flattening of the heart rate during incremental exercise to determine a deflection point equivalent to the anaerobic threshold. Although there is no full agreement in literature about the reliability and validity of this method, Bodner and Rhodes (5) as well as Hofmann and Pokan (6) gave a comprehensive overview of its value.

Interestingly, a significant number of tests show a linear or even an inverted time course of the HRPC. Although most young healthy subjects (~86%) showed a regular deflection of the HRPC, in this homogenous group of 227 (23 ± 4 years) trained male sports students an inverse deflection was found in in 7.9% cases and a linear time course was found in 7.9% of subjects in this study (1). Additional information is sparse and only one study by Heber et al. (7) presented data on HRPC deflection in 128 patients during cardiac rehabilitation, however did not show the distribution among subjects. Pokan et al. (8) was the first to show increasing atypical HR curves in older patients suffering from heart disease. Converse to healthy subjects, most patients (86 %) showed an inverse deflection in the exercise tests administered ~57 days after myocardial infarction. A regular deflection was only found in 4% and a linear time course in 10%.

Underlying physiological regulations such as a parasympathetic or sympathetic influences, as well as the relationship to left ventricular function on the degree and direction of HRPC deflection, have been investigated in several studies compiled in a review (6). The most plausible and actually valid explanation for the different HRPC patterns was found for β1-receptor sensitivity shown by our study group (9). This hypothesis was supported most recently by results from cardio-pulmonary exercise testing in individuals with type 1 diabetes (10) where the degree of HRPC deflection was significantly lower in individuals with type 1 diabetes compared to matched controls. These authors concluded, that constantly elevated HbA_1c_ levels and concomitantly elevated catecholamine levels and/or inflammation induced chronic stress impairs β1-receptor sensitivity, which alters the degree and direction of the HRPC. Such a pathophysiological value of the HRPC can also be found in patients after myocardial infarction, who frequently present an upward deflection in incremental exercise tests (11). A normalization of the HRPC was found with increasing cardiorespiratory fitness after a 1 year exercise-based cardiac rehabilitation program independent from medication (7).

As the pattern of the HRPC has substantial consequences for the prescription of exercise intensity it was argued that using the same fixed percentage of maximal heart rate (HR_max_) will result in different workloads with respect to the anaerobic threshold (12). Subjects with an atypical HRPC are prone to overload compared to subjects with regular HRPC's (2, 13).

A well-examined factor influencing HR_max_ is age. From the age of 18 to 50 years, HR_max_ was shown to decrease linearly by 0.7 beats·yr^−1^, with a smaller decrease in younger adulthood (14). Several reasons for this decrease in HR_max_ with age have been discussed, although no definite conclusion has been drawn yet. Beside the age associated decline in cardiac autonomic function (15) and a reduction of intrinsic heart rate (HR_int_) with age (16), one main reason may be a reduced β-adrenoreceptor sensitivity or density with cardiac aging. Studies examining the cardiovascular effects to graded isoproterenol and propranolol infusions showed a reduced β-adrenergic responsivity with advancing age (16–18). This could be addressed to age associated reductions in HR_max_ (16).

Regarding the influence of exercise, even high levels of daily physical activity had no effects on heart rate response with respect to age (15). On the other hand, Rogers et al. (19) showed no decrease in maximum heart rate in well-trained master endurance athletes in a period of 8 years of continues training compared to sedentary controls. This is in line with other results, which showed a slower rate of decline in HR_max_ in people with higher cardiorespiratory fitness (20). Nevertheless, cardiorespiratory fitness declines non-linear up to 45 years followed by an accelerated decline with increasing age (21).

These well-known age-related changes in HR_max_ are therefore also suggested to alter the pattern of the HRPC during aging. Hence, the aim of this study was to examine the effect of aging on the deflection of the HRPC in a large cohort of healthy male and female subjects. We hypothesized that the number of atypical HRPC's is increasing with age but different between sexes and modulated by exercised performance.

## Methods

This study was performed within the “HEALTHeBIKES” project in order to classify potential users by their HR response to exercise and to investigate whether programming a HR based e-bike control is depending on age. The study has been approved by the Ethics Committee of the local University.

**Figure 1:**
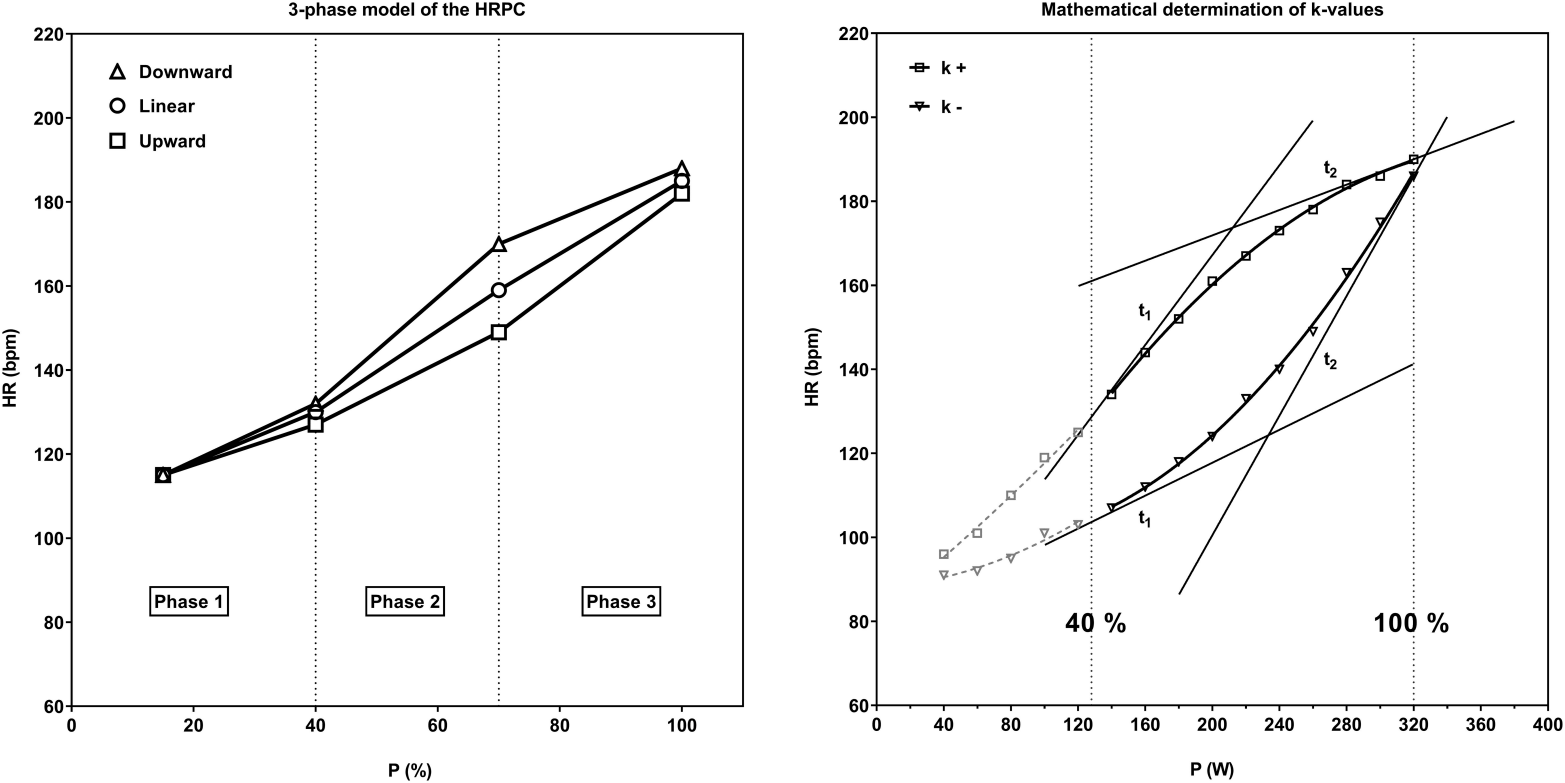
Schematic 3-phase model of the HRPC from incremental cycle ergometer exercise with regular downward deflection, linear time course and upward deflection (1). Mathematical determination of *k*-values from HRPC's with downward (*k*+) and upward deflection (*k*–). The degree and the direction of the HRPC deflection (*k*) was calculated from the difference of angles of the tangents (t_1_, t_2_) from a polynomial best fit between 40 and 100% of P_max_ (8).

Heart rate and performance data from 30,000 cycle ergometer tests carried out for performance diagnostic, health preventive or medical reasons between 2004 and 2017 were obtained. Generally, this tests last about 15 min and individuals were encouraged not to do any vigorous activity the day before. Finally, 2,980 healthy men (age: 51 ± 15 years., range 13–87 years) and 1,944 women (age: 52 ± 14 years, range from 13 to 89 years) who performed the same test protocol were included in the study. Only tests with correct HRPC presentation (no outliers or interruptions of HR recordings) and a HR_max_ within 15% of the expected HR_max_ calculated as 210-age were included in our analysis. The test protocol was uniform and independent from age, gender and performance and was applied according to the recommendations of the local Society of Cardiology (22). All ergometer tests started at 20 W and power was increased in uniform 20 W increments per minute up to exhaustion. In our retrospective analysis, we categorized the tests into eight age groups starting with ≤20 years up to >80 years (Table 1). The degree and the direction of the HRPC deflection was calculated by a second-degree polynomial fit curve (least error square) (8) from the mean HR of each single load-step between 40 and 100% of P_max_ (Figure 1). From this quadratic function the slopes (k_1_ and k_2_) of the tangents (t_1_ and t_2_) in the two HR-points of the curve corresponding to 40 and 100% P_max_ were calculated. With the slopes of these two tangents the type of deflection was determined by using the factor *k* (*k* = (*k*_1_-*k*_2_) / (1 + $k_{1^{*}}$k_2_)). *k*-values were classified as downward deflection *k*+ (*k* > 0.1) (regular), linear *k*0 (−0.1 ≤ *k* ≤ 0.1) and upward deflection *k*– (*k* < −0.1) (atypical) (Figure 1).

**Table 1:** Number of tests (*N*) and mean ± SD age, BMI, body mass (BM) and %P_max_ of each age group in men and women.

**Men (2,980)**	**≤20**	**21–30**	**31–40**	**41–50**	**51–60**	**61–70**	**71–80**	**>80**
*N*	105	236	455	651	670	555	271	37
Age (years)	17.8 ± 1.8	26.0 ± 2.8	36.1 ± 2.9	45.8 ± 2.8	55.3 ± 2.9	65.3 ± 2.9	74.3 ± 2.5	83.2 ± 1.8
BMI (kg/m^2^)	22.3 ± 2.8	24.2 ± 3.8^*^	25.5 ± 3.4^*^	26.4 ± 3.3^*^	26.8 ± 3.6	26.6 ± 3.3	26.7 ± 3.3	26.1 ± 3.2
BM (kg)	72.4 ± 10.7	79.2 ± 12.9	83.2 ± 12.2	85.0 ± 11.5	85.4 ± 12.5	82.5 ± 11.5	81.1 ± 11.6	77.2 ± 10.6
%P_max_ (%)	108 ± 18	114 ± 21	124 ± 24^*^	120 ± 21	122 ± 20	119 ± 22	112 ± 19^*^	105 ± 18
**Women (1,944)**								
*N*	33	151	207	429	505	424	171	24
Age (years)	18.2 ± 1.8	26.2 ± 2.7	36.4 ± 2.8	45.9 ± 2.9	55.2 ± 2.9	65.1 ± 2.8	74.4 ± 2.6	83.4 ± 1.9
BMI (kg/m^2^)	22.0 ± 2.8	22.1 ± 3.7	23.0 ± 3.9	24.0 ± 4.3^*^	25.2 ± 4.2^*^	25.8 ± 3.9	25.8 ± 4.0	25.5 ± 3.4
BM (kg)	62.4 ± 9.3	61.6 ± 11.1	64.3 ± 11.6	66.5 ± 12.4	68.5 ± 12.3	68.5 ± 10.7	68.1 ± 11.2	65.1 ± 8.8
%P_max_ (%)	125 ± 18	129 ± 18	127 ± 23	126 ± 21	119 ± 19^*^	111 ± 20^*^	101 ± 18^*^	93 ± 17

* *Significant different compared to the previous younger age group*.

*N denote the number of analyzed tests*.

%P_max_ denotes P_max_ in percent of age predicted power (P_pred_) which was calculated for men: P_pred_ = 6,773 + 136,141_*_BS-0.916_*_BS_*_age and women: P_pred_ = 3,933 + 86,641_*_BS-0.346_*_BS_*_age whereby BS denotes body surface calculated as: BS = 0.007148_*_BW (kg)^0.425^·H (cm)^0.725^ (23).

Furthermore, P_max_ in percent of age predicted power (%P_max_) was calculated using a standard equation (Table 1) from the local cardiological society (22). To assess the effect of performance on HR deflection and HR_max_ we compared subjects with low (P_low_: 25%-quartile) and high (P_high_: 75%-quartile) performance (%P_max_) within each age group.

### Data Analysis

Data Analysis was performed using GraphPad Prism 7 (GraphPad Software, San Diego, CA). For confirmation of normality, the Shapiro-Wilk normality test was used. Repeated measures ANOVA with Dunn's multiple comparison test was used to compare *k*-values, HR_max_, P_max_, %P_max_, and BMI between age groups. Linear regression analyses were performed to prescribe the age-related changes of the HRPC and the absolute *k*-values. Data are presented as means ± SD. Statistical significance was set at *p* < 0.05.

## Results

**Figure 2:**
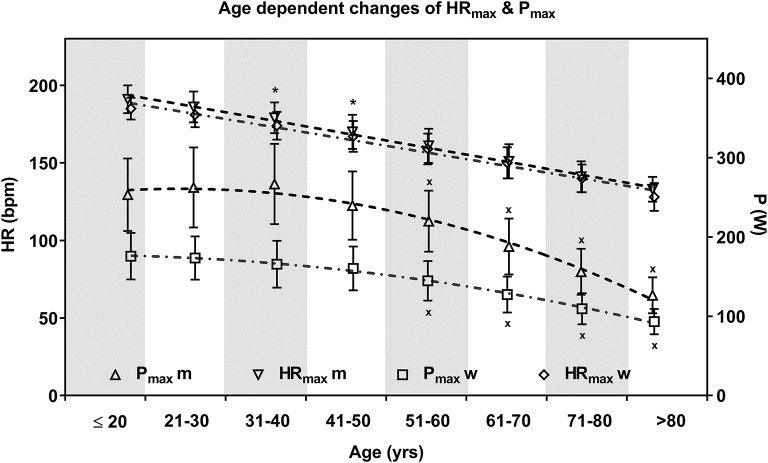
Mean ± SD maximum heart rate (HR_max_) and power (P_max_) of the age groups in men and women. Age course of HR_max_ is described via linear regression and P_max_ via quadratic function. Men and women were significantly different for P_max_ within each age group but not for HR_max_ except for two age groups. Aging was significantly related to decreasing HR_max_ within each age group and decreasing P_max_ in the age groups older 50 years in men and women. * significant different HR_max_ between men and women; ^x^ P_max_ significant different from repeated measure ANOVA (*p* < 0.05).

The number of analyzed tests as well as the mean age, BMI and body mass of each age group are shown in Table 1. Maximum HR decreased linearly with age (*m*: HR_max_ = 212–0,93_*_age, *r* = 0.82; *w*: HR_max_ = 205–0.85_*_age, *r* = 0.80) from 191 ± 9 bpm (*m*) to 185 ± 7 bpm (*w*) in the youngest age group to 133 ± 8 bpm (*m*) and 128 ± 9 bpm (*w*) in the oldest age group (Figure 2). The mean decrease per decade of both groups was 8.2 ± 1.9 bpm, whereas the HR_max_ decrease was less in subjects younger than 50 years (6.5 ± 1.3 bpm) compared to subjects older than 50 years (9.5 ± 1.3 bpm). Correlation analyses of HR_max_ and age-predicted maximum heart rate calculated from Tanaka et al. (24) (HR_max_ = 208–0.7_*_age), were comparable and significant (*r* = 0.81).

Maximum power was unaffected by age up to 50 years in men (P_max_ = 253 ± 49 W) and women (P_max_ = 165 ± 29 W) and decreased significantly in the older age groups. The lowest P_max_ (*m*: 126 ± 22 W; *w*: 93 ± 16 W) was present in the oldest age groups (>80 years) (Figure 2). Mean %P_max_ varied between age groups and decreased with increasing age. Except for the oldest female age group, mean %P_max_ was above 100%, which means normal with respect to the given guideline norms (22) in every age group.

### Age Dependent Effects on HRPC

**Figure 3:**
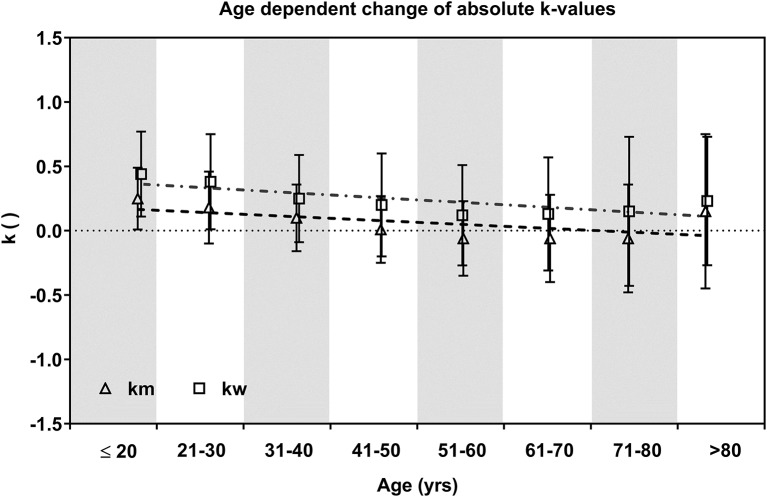
Mean ± SD *k*-values of age groups in men and women. Dashed (*m*) and dot dashed (*w*) lines show the decrease via linear regression.

The HRPC pattern significantly changed with increasing age both in men and women indicated by a significant decrease (ANOVA) of *k*-values in age groups >31 years (*m*) and >41 years (*w*) compared to subjects ≤ 20 years. In the older age groups *k* was not significantly different in men from 51 to 80 years and in women from 41 to >80 years. The highest *k*-values (*k*_m_ = 0.25 ± 0.24 / *k*_w_= 0.44 ± 0.33) were found in the youngest age groups (≤20 years). *k*-values were significantly higher in women compared to men. In men mean values even reached negative values in age groups older than 50 years which indicates an increasing number of atypical inverted HRPC's (Figure 3).

**Figure 4:**
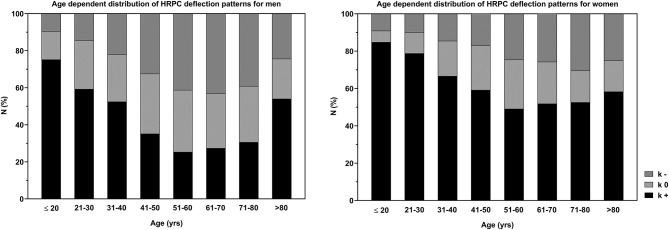
Distribution of the HRPC deflection pattern (regular downward deflection: *k*+, atypical upward deflection: *k*–, linear heart rate deflection: *k*0) for all age groups in men and women.

Figure 4 shows the distribution of *k*-values, categorized as regular downward deflection (*k*+) as well as atypical upward (*k*–) and linear HRPC deflection (*k*0). The number of heart rate curves with upward deflection increased from 10 to 43% in men ≤20 to 70 years, but decreased slightly between 71 and 80 years and substantial in the oldest age group >80 years. In women, the number of atypical curves was smaller compared to men and increased from 9 to 30% for ≤20 to 80 years with a smaller increase from 51 to 80 years, but also decreased in the age group >80 years. The increase of atypical curves with age shows a linear trend up to 80 years. Conversely, the number of cases presenting a regular downward deflection decreased from 75 to 52% (*m*) and 85 to 49% (*w*) for subjects ≤20 to 60 years and increased slightly from 61 to >80 years in both groups with a large increase in men >80 years. The number of linear curves was less affected by age.

### Exercise Performance and HRPC

**Figure 5:**
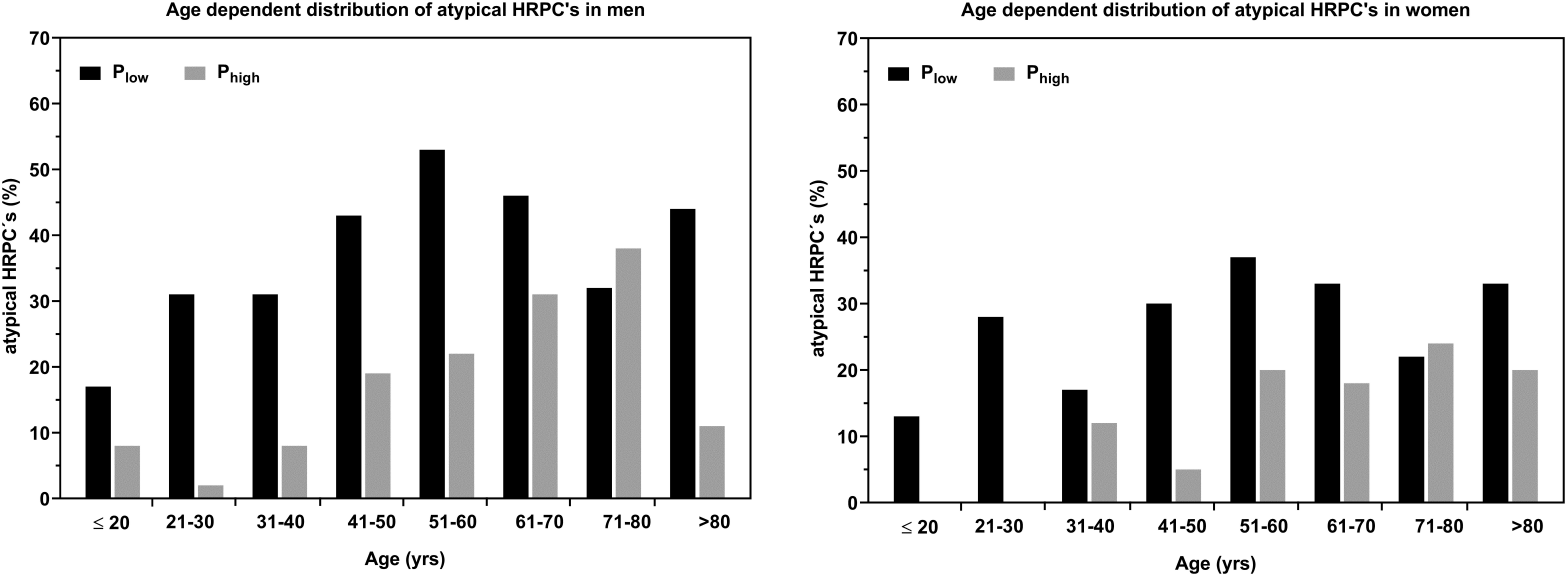
Age dependent relative number (%) of HRPC's with atypical upward deflection for high (P_low_) and low (P_high_) exercise performance groups (25% respectively 75%-quartile %P_max_) for men and women.

Assessment of 25 and 75%-quartile of %P_max_ revealed a mean limit of all age groups for the lower quartile (P_low_) at 102 ± 6% (*m*) / 103 ± 12 (*w*) %P_max_ and for the upper quartile (P_high_) at 128 ± 6% (*m*) / 128 ± 14 (*w*) %P_max_. HR_max_ was lower in P_low_ compared to P_high_ of the single age groups, whereby HR_max_ was significantly different in men between >40 to 70 years and in women between >30 to 70 years. The overall mean difference was 6 ± 2 bpm (*m*) and 7 ± 2 bpm (*w*). Higher *k*-values were found in P_high_ in age groups >20 to 70 years (Δk = 0.15 ± 0.04) in men and <20 to 60 years (Δk = 0.16 ± 0.08) in women. *k*-Values were significantly different for male subjects between >20 to 60 years and for female between >20 to 30 and >40 to 50 years. The number of curves with atypical upward deflection was higher in P_low_ compared to P_high_ in men and women except for the age groups >70 to 80 years. The increase of atypical curves was delayed to older age in P_high_ (Figure 5).

## Discussion

Our analysis of a large cohort of age and performance heterogenous healthy male and female subjects confirm previous results that the HRPC in incremental exercise tests is neither uniform nor linear (1, 2) and the well-known decrease of HR_max_ with age. For the first time we show significant age related changes of the pattern of the HRPC with an increase in the number of atypical curves with an upward inflection of HR. These changes were significantly different between men and women and exercise performance had some minor effects on HR_max_ and the pattern of the HRPC.

Comparable to our older results in young healthy male trained subjects (1), young subjects in our study presented the highest number of regular HRPC's with a downward deflection, suggested as “normal.” However, our study group had a lower number of regular HRPC's in subjects in the same age group. Toward an age of 50 years changes in the degree of the HRPC deflection and the number of atypical curves were stronger compared to >50 years. Sparse studies showed that patients with cardiovascular disease mostly displayed an upward deflection of the HRPC (7, 11), prescribed also in people with type 1 diabetes (10), however no studies in age heterogenous healthy subject have been presented so far.

### ß1-Receptor Sensitivity

The decrease in HR_max_ with age was comparable with recent studies (14, 24). As the decrease of HR_max_ with age was associated with β-receptor insensitivity (16–18) and/or reduction in receptor density (25) this suggests a causal relation between the changes of the HRPC deflection with age and β1-adrenergic receptor function. A reduced receptor sensitivity was shown to blunt the heart rate response at moderate exercise intensity where catecholamine levels are still low (26). At higher intensities catecholamine levels increase exponentially and thus receptors can be stimulated, increasing HR disproportionally compared to the smaller increase at low to moderate exercise intensity. This phenomenon was prescribed to cause an upward deflection of the HRPC (9)

### Sex Differences

The overall number of atypical curves (*m*: 33%, *w*: 21%) and the degree of the HRPC deflection was different for men and women, who presented a comparable decrease of k, but women had significantly higher values throughout the whole age span. These sex differences in the deflection of the HRPC can also be assigned to varied responses of men and women to β-adrenergic stimulation shown by isoproterenol infusion (23, 27). Men had a greater age-associated decrease in inotropic and chronotropic responses to catecholamines compared to women (23). In addition, β-adrenergic responsiveness was higher in young and older women compared to male reference groups (27). Referring to the causal relation between the deflection of the HRPC and β1-adrenergic receptor sensitivity, this may explain the higher number of regular HRPC's in women. Beside ß1-adrenoceptor sensitivity women were shown to have differences in autonomic functions (28) although intrinsic HR may not be that important (29).

### Performance Differences

Classification into low- and high-performance groups revealed lower *k*-values in 5 (*m*) respectively 6 (*w*) age groups in P_low_. The number of atypical curves in each single age group was substantially higher in P_low_ compared to P_high_ except for male and female age groups >70 to 80 years. Overall the number of atypical curves was 21% (*m*) and 16% (*w*) higher in P_low_ compared to P_high_, indicating a delayed onset of physical frailty in the P_high_ (30). The distribution of atypical curves in the age groups >70 to 80 years might be due to a general higher number of subjects in good condition in this age group, however relative P_max_ do not support this suggestion. Anyhow, this group is suggested as healthy “survivors” who already exceeded the age of expected healthy life years which might lower the physiological differences between the high and low performance group.

Lehmann et al. (31) showed that higher performance was related to better β1-receptor sensitivity which supports our data showing that subjects with higher performance presented a higher number of regular curves in line with earlier results from our working group (9). Additionally, several studies showed improved β-adrenergic responsiveness after 12 weeks treadmill training in rats and reversed β-adrenergic disfunction due to restored cardiac receptor density after exercise (32). Also aerobic exercise training was shown to reduce circulating catecholamine's (33), which counteracts reduced β-adrenergic density due to overactivation of the sympathetic nervous system and concomitant chronic catecholamine stimulation (34). Thus, sensitization of the β1-receptor through regular exercise might normalize atypical HRPC provoked by chronic stress due to diseases such as type 1 diabetes (10) or cardiovascular disease (11) as shown recently (7).

Mean exercise performance of all single age groups, except for the oldest age group in women, was higher compared to the age predicted maximum power output which may be classified “normal.” Further, maximum ergometer power output was constant up to 50 years which is in line with earlier findings (21), but different to guideline values, which present a continuous decrease with age (22, 35). The P_max_ decline was independent from HR_max_ as already shown (36). HR_max_ though was lower in the low compared to the high-performance group. This might be due to higher performance motivation of well-trained subjects, but might also be caused by a higher O_2_ extraction and concomitant higher cardiac output due to a higher muscular aerobic capacity in the high-performance group (19). Our results are in line with other studies who showed reduced decline of HR_max_ in people with high cardiorespiratory fitness and confirm that decreasing HR_max_ with aging is to a certain extent preventable by higher performance levels (19, 20). Anyway, mean P_max_ of our cohort up to 50 years was 253 ± 49 W in men and 165 ± 29 W in women.

### Exercise Prescription

From a practical point of view the heterogenous character of HRPC's has some consequences on exercise prescription when using fixed percentage of HR_max_, already discussed in detail by our study group (2, 12) which was supported recently by Iannetta et al. (37). We could nicely show that the same relative intensity of 85% HR_max_ gave different workloads when related to the anaerobic threshold and this effect became even stronger with ß_1_-recepter antagonism application (13). The calculation of a fixed percentage consequently leads to an underestimation of the workload in regular HRPC's but an overestimation in upward deflecting atypical curves. The use of such a %HR_max_ method may overestimate training heart rate by at least 5–10% and up to 40% in single cases (12). Underestimation might cause absence of desired training effects, but overestimation of the workload presents some risks and could have major consequences in subjects suffering from a chronic disease. Iannetta et al. (37) supported this earlier results and concluded that “contemporary gold-standard methods for exercise prescription based on fixed-percentages of maximum values conform poorly to exercise intensity domains and thus do not adequately control the metabolic stimulus.”

Due to the changes of the HRPC pattern with aging, an age dependent prescription of exercise intensity needs to be developed as usual linear equations are not appropriate. The given guideline range of 77–95% HR_max_ (38) to calculate the upper target training limits represent a wide spectrum of cardio-respiratory and metabolic responses (39), so to fulfill the needs for an individualized approach, individual thresholds need to be determined (37).

Some limits of the study are to be mentioned. The generalizability of our study is limited. Exercise tests were performed in external institutions in terms of medical screenings. For our analyzes only apparently healthy individuals with no recorded medication were used, but insufficient documentation and comorbidity cannot be fully excluded. Additionally, in this retrospective study the actual activity level or training regime was not controlled directly. Further the exercise protocol was independent from the individual performance. The number of HR points for analysis were therefore smaller in individuals with low P_max_. This causes less accuracy in the calculation of absolute k-values, but does not affect the classification of deflection types. Talking about the strengths, to our knowledge this is the first study investigating the effect of age on the HRPC time course. The great number of subjects included allowed to draw some general conclusions in this large cohort of healthy men and women.

## Conclusion

The number of atypical HRPC's presenting an upward deflection in a maximal incremental cycle ergometer exercise test increases with age. These changes were influenced by age, sex, and performance, where subjects with higher performance presented less atypical HRPC even at older age. Because the number of atypical curves substantially increased with age we suggest to modify linear percent HR_max_ exercise prescription models as the chance to overestimate training intensity increases with age. Furthermore, the determination of the HRPC deflection is suggested to give additional information regarding the health and performance status of subjects. Further research should focus on more detailed analysis of different subgroups such as patients, suffering from different chronic diseases, as shown nicely by Heber et al. (7).

## Data Availability Statement

The datasets generated for this study are available on request to the corresponding author.

## Ethics Statement

The studies involving human participants were reviewed and approved by Ethic Committee University of Graz, Universitätsplatz 3, 8010, Graz. Written informed consent to participate in this study was provided by the participants' legal guardian/next of kin.

## Author Contributions

PH, RM-O, HT, and PB conceived and designed the experimental plan. PB and AB analyzed the data. PB and PH drafted the manuscript. MF, PH, HH, and PB refined and approved the final manuscript. All authors proofread and accepted the final version of the manuscript.

### Conflict of Interest

The authors declare that the research was conducted in the absence of any commercial or financial relationships that could be construed as a potential conflict of interest.
